# Triptolide targets PPP2CA/ITGA5 axis to suppress lactate-driven ovarian cancer progression

**DOI:** 10.1186/s13020-025-01174-2

**Published:** 2025-08-06

**Authors:** Ling Ding, Wutao Chen, Cenxin Luo, Nathaniel Weygant, Yi Lai, Dan Ru, Hengan Liu, You Wang, He Li

**Affiliations:** 1 Traditional Chinese Medicine Department, School of Medicine, Renji Hospital, Shanghai Jiao Tong University, 160 Pujian Road, Shanghai, 200127 China; 2 Department of Obstetrics and Gynecology, School of Medicine, Renji Hospital, Shanghai Jiao Tong University, 160 Pujian Road, Shanghai, 200127 China; 3 Shanghai Key Laboratory of Gynecologic Oncology, School of Medicine, Renji Hospital, Shanghai Jiao Tong University, 160 Pujian Road, Shanghai, 200127 China; 4 Shanghai University of Traditional Chinese Medicine, 1200 Cailun Road, Shanghai, 201203 China; 5 Academy of Integrative Medicine, Fujian University of Traditional Chinese Medicine, Fuzhou, 350122 China; 6 Department of Head and Neck Surgery, School of Medicine, Renji Hospital, Shanghai Jiao Tong University, 160 Pujian Road, Shanghai, 200127 China; 7 Faculty of Medicine, The University of Queensland, St Lucia, QLD 4072 Australia

**Keywords:** Ovarian cancer, *PPP2CA*, *ITGA5*, Lactate, Triptolide

## Abstract

**Background:**

Triptolide, the active compound of *Tripterygium wilfordii*, exhibits broad anti-tumor activity. This study explores *PPP2CA* dysregulation in ovarian cancer (OC) progression via lactate production and evaluates Triptolide’s potential to regulate this process.

**Methods:**

We used patient-derived xenograft (PDX) models, cell proliferation, and migration assays to assess lactate’s impact on OC progression. CRISPR-Cas9 was applied to knock out *PPP2CA*, examining its effect on lactate production and tumor progression. RNA-seq analyzed transcriptomic changes post-*PPP2CA* knockout. The *PPP2CA-ITGA5* axis was validated using xenografts, immunofluorescence, immunohistochemistry staining and western blot. Exosome isolation and co-culture experiments with tumor cells and human peritoneal mesothelial cells (HPMCs) investigated *ITGA5*’s role in migration. Finally, patient-derived organoids, xenograft tumor model, and lactate assays assessed Triptolide’s reversal effect on *PPP2CA* dysregulation-driven OC progression.

**Results:**

We found that *PPP2CA* dysregulation significantly promotes OC proliferation, migration, and tumorigenesis by enhancing *YAP* nuclear translocation and upregulating *ITGA5/ITGB1*. *PPP2CA* dysregulation led to *ITGA5* upregulation, where *ITGA5*, as part of the *integrin α5β1* heterodimer, plays a key role in driving OC migration. Exosomal *ITGA5* facilitates OC metastasis to the HPMCs. Triptolide effectively inhibited patient-derived organoid growth and reduced lactate production in OC cells. By suppressing *ITGA5*, Triptolide reversed cancer progression and restored tumor-suppressive effects in a *PPP2CA*-knockout xenograft model.

**Conclusion:**

Our study reveals that Triptolide effectively inhibits OC progression by targeting the *PPP2CA-ITGA5* axis, mitigating lactate-driven metabolic reprogramming.

**Graphic Abstract:**

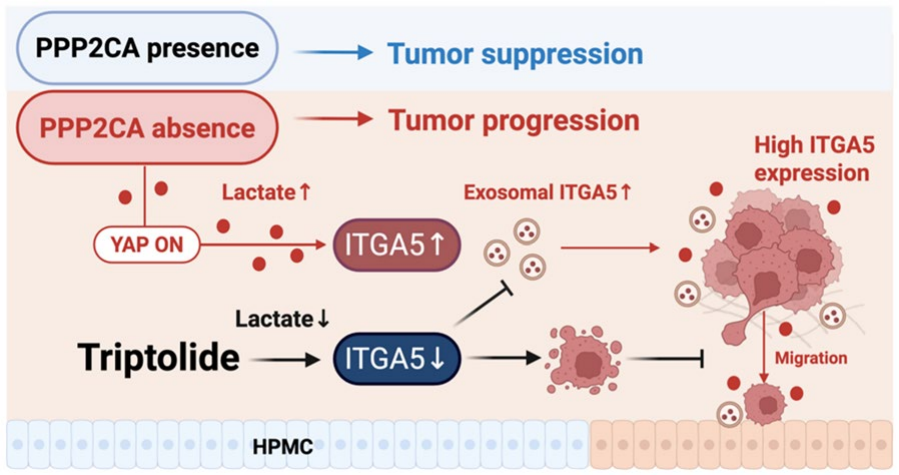

**Supplementary Information:**

The online version contains supplementary material available at 10.1186/s13020-025-01174-2.

## Highlights

1. Lactate is a key driver of OC progression, with *PPP2CA* facilitating its production.
2. *PPP2CA* dysregulation promotes expression of an *Integrin α5*-dominant *β1* heterodimer which promotes tumorigenic and the formation of a pre-metastatic niche.

3. Triptolide effectively targets *ITGA5*, suppressing tumor progression in OC.

**Supplementary Information:**

The online version contains supplementary material available at 10.1186/s13020-025-01174-2.

## Background

Ovarian cancer (OC) is a leading cause of cancer-related mortality in women due to its covert dissemination and metastasis. OC cells spread through peritoneal fluid, making their stealthy progression difficult to treat. Due to the lack of effective early diagnostic tools, over half of OC cases are diagnosed at advanced stages with metastasis [1] and effective treatment options for managing late-stage OC remain limited [2]. There is an urgent need to explore more effective therapeutic strategies and to identify novel therapeutic targets to improve OC patient outcomes.

Glycolysis is the predominant energy metabolism pathway in cancer cells, with lactate being the final product. In the tumor microenvironment (TME), lactate functions as an intercellular signaling metabolite. Elevated lactate levels not only activate oncogenic pathways but also facilitate a mutualistic relationship between tumor and stromal cells, promoting glycolytic flux and lactate accumulation in a positive feedback loop. This process is critical for cancer progression, underscoring lactate’s prominent role in tumor metabolism [3, 4]. Several studies have demonstrated that lactate is a regulator of the OC TME and disease progression. OC cells produce excessive lactate, which remodels tumor-associated macrophages (TAMs) into the M2 phenotype via the lactate sensor/receptor *GPR132* [5]. This phenotypic switch promotes an immunosuppressive environment conducive to tumor growth. Additionally, lactate degrades the tumor suppressor *STAT1* through the proteasomal pathway, acting as a negative regulator of the IFNα-*STAT1* signaling axis in OC cells. This degradation limits the anti-tumor effects of IFN-α, thereby weakening immune defenses against OC [6]. The prognostic significance of lactate in OC is supported by studies demonstrating that a gene signature based on lactate metabolism can function as an independent prognostic indicator for predicting outcomes in serous OC [7]. Clarifying the drivers of lactate overproduction and its mechanistic role in OC progression may be essential for developing targeted therapies. Overall, while lactate’s impact on OC is recognized, the specific mechanisms underlying its influence remain insufficiently explored.

Protein phosphatase 2A (*PP2A*) is a pivotal serine/threonine phosphatase, comprising a holoenzyme complex with a scaffolding (A), regulatory (B), and catalytic (C) subunit, is pivotal in cellular regulation and widely acknowledged as a tumor suppressor in various solid malignancies [8, 9], as the primary catalytic subunit responsible for dephosphorylation, *PPP2CA* plays a pivotal role in cancer progression, with its downregulation closely linked to malignancy. For instance, low *PPP2CA* expression serves as an independent prognostic marker for poor outcomes in colorectal cancer [10]. In prostate cancer, *PPP2CA* downregulation is linked to castration resistance and the activation of epithelial-mesenchymal transition (EMT), contributing to a more invasive tumor phenotype, restoration of *PPP2CA* activity can reverse EMT, significantly suppressing tumor growth and metastasis [11]. However, the role of *PPP2CA* in OC remains undefined. Recent investigations reveal that tumor cells exhibit the ability to alternate between glycolytic and oxidative phosphorylation (OXPHOS) dependencies, a phenomenon termed metabolic plasticity. *PP2A* functions as an early-response sensor to energy stress, where the combined application of caloric restriction and metformin, an OXPHOS inhibitor, necessitates *PP2A* as a crucial mediator for targeted therapeutic effects [12]. In tumor cells, eukaryotic elongation factor-2 kinase has been reported to promote glycolysis by restricting *PP2A* synthesis [13]. In physiological settings, liver-specific *PPP2CA* knockout was shown to enhance glycolysis and accelerate the termination of liver regeneration [14]. Given its established role in cancer biology and growing evidence implicating *PP2A* in glycolysis control, the specific involvement of *PPP2CA* in glycolytic reprogramming and metabolic adaptation in OC warrants further investigation.

Solid tumors predominantly rely on intercellular adhesion, with integrins regulating both cell-to-cell and cell-to-matrix interactions, thus playing a crucial role in tumor progression and metastasis. Integrins are often carried by exosomes as functional molecules, playing a critical role in intercellular signal transmission and communication. OC exosomes can overcome invasive barriers of the pre-metastatic niche. For instance, exosomes carrying specific miRNAs remodel mesothelial cells, enhancing peritoneal permeability [15]. This intercellular crosstalk facilitated by exosomes promotes tumor homing [16, 17]. Current research indicates that *integrin α5β1* is highly expressed in human peritoneal mesothelial cells (HPMCs) of OC patients [18]. *Integrin α5β1* is a heterodimer consisting of *α5* and *β1* subunits, primarily mediating cell adhesion to the extracellular matrix (ECM) or other cells. Upon ligand binding, integrins undergo conformational changes, connecting to cytoskeletal proteins, and activating downstream signaling pathways. This process includes inside-out signaling, where intracellular signals regulate the integrin’s ability to bind to ECM ligands, and outside-in signaling, which drives cytoskeletal reorganization. This bidirectional signaling pathway modulates cellular responses to external stimuli, such as adhesion, migration and survival [19–21].

Triptolide, the principal bioactive component of *Tripterygium wilfordii*, has been widely recognized for its broad-spectrum antitumor properties. Some studies have demonstrated its therapeutic efficacy in various malignancies, including breast cancer, lung cancer, cervical cancer [22–24]. Triptolide exerts potent inhibitory effects on tumor cell proliferation at extremely low concentrations and induces apoptosis. In OC, Triptolide suppresses tumor progression by targeting *NF-κB* activation and subsequently downregulating *HER2* expression [25]. Additionally, Triptolide promotes apoptosis in OC cells through multiple mechanisms, such as modulating the expression of apoptosis-related genes *Bcl-2/Bax*, activating caspases while inhibiting the *NF-κB* signaling pathway [26], and regulating the expression of heat shock proteins (*HSP*)-*27* and *HSP-70*, leading to apoptosis in various OC cell lines [27]. Furthermore, Triptolide has been reported to overcome chemoresistance in OC by increasing intracellular reactive oxygen species (ROS) levels [28]. However, the precise mechanisms underlying Triptolide’s role in OC remain inadequately elucidated.

In this study, we demonstrate that Triptolide effectively suppresses OC growth and migration by targeting the *PPP2CA-ITGA5* axis, thereby mitigating lactate-driven metabolic reprogramming. Specifically, Triptolide inhibits *ITGA5* expression, reducing OC cell migration and exosome-mediated pre-metastatic niche formation.

## Materials and methods

### Cell culture and transfection

OC cell lines SKOV3, OVCAR8 and mouse-derived ID8 were cultured in DMEM high-glucose medium (HyClone, SH30243) with 10% FBS and 1% antibiotics, and authenticated by STR profiling. SKOV3-Luc cells were generated by transfecting SKOV3 cells with a luciferase reporter plasmid containing puromycin resistance. For stable transfection, shRNAs targeting *ITGA5*, *ITGB1*, *ITGA5/ITGB1*, and an *ITGA5*-eGFP overexpression plasmid, along with control plasmids, were designed by Genomeditech (Shanghai, China). Positive cells were selected using blasticidin (5 μg/mL) and puromycin (3 μg/mL).

For *PPP2CA* knockout, sgRNA (5'-3' acgtgcaagaggttcgatgt) was cloned into the LentiCRISPRv2 vector (Addgene, 52,961). Lentiviral particles were produced by transfecting HEK293FT cells with psPAX2 (Addgene, 12,260) and pMDG.2 (Addgene, 12259) using EZ trans (Life-iLab, AC04L091). SKOV3 cells were infected for 24 h and selected with puromycin (3 μg/mL) for stable knockout.

### Colony formation

Cells were plated at a density of 500 cells per well in 12-well plates. Sodium oxamate (10 mM, Selleck, S6871) or Stiripentol (100 µM, Selleck, S5266) was added the following day. Colony formation was monitored daily for approximately 10 days. Once colonies exceeded 50 cells, the culture was halted. Cells were washed with PBS, fixed in 4% paraformaldehyde, stained with crystal violet, and rewashed before imaging and colony counting for analysis.

### Wound healing assay

Cells were seeded in 12-well plates and cultured until they reached 80–90% confluence. A scratch was then made using a 200 µL pipette tip to create a consistent wound across the cell monolayer. Images were taken immediately at 0 h (10 × magnification) to capture the initial wound. Drug treatments were applied immediately after the scratch was made. After 24 h, images of the wound healing were taken. The migration percentage for each group was calculated by comparing the width of the scratch at 24 h to the initial width at 0 h within the same well, with 0 h defined as 0% healing. The residual wound width was normalized to the initial width at 0 h for each group, allowing for accurate comparison of migration rates between groups while accounting for initial wound size variability.

### Transwell assay

Different cells and their control groups were resuspended in serum-free medium and counted (200 µL, 3 × 10^4^ cells). The suspension was added to 8.0 µm pore membrane insert (Corning, 3422), with the lower chamber filled with complete medium containing 10% FBS. The cells were fixed with 4% paraformaldehyde after 24 h. Crystal violet staining was applied to both sides of the insert, and non-migrated cells on the upper membrane were wiped off. After drying, 10 × magnification images of the migrated cells were captured, followed by counting and statistical analysis.

### Isolation of HPMCs

HPMCs were isolated from omental tissue obtained from healthy donors undergoing laparoscopic surgery, following the protocol by Cai et al. [29] In brief, approximately 10 cm^2^ of omental tissue was washed twice with PBS and cut into smaller Sects. (2–3 cm^2^). The sections were incubated in 0.25% trypsin at 37 °C for 30 min. The trypsinized solution containing HPMCs was centrifuged at 500 × g for 5 min, and the pellet was plated in DMEM with 10% FBS for culture.

### Generation of OC organoids

Tumor tissue from OC patients was obtained during surgery, rinsed with DPBS, and trimmed to remove non-tumor components. The tissue was minced into fragments and digested in Tumor Tissue Digestion Solution (bioGenous, K601003) at 37 °C with vigorous shaking. When digestion was complete, the mixture was passed through a 100 µm strainer, and the cells were pelleted by centrifugation at 300 × g for 3 min. After red blood cell lysis, cell pellet was mixed with Matrigel (Mogengel, 082755) and plated into 24-well plates (50 µL per well). After 30 min of incubation, OC organoid medium (bioGenous, K2168) was added, the culture medium was refreshed every other day, and the organoid growth was monitored and recorded.

### Organoid growth assay

Organoids were isolated and seeded into 96-well clear-bottom black plates (Greiner, 655090) at 100 organoids per well. After several days of growth, when organoids were nearing maturation, different concentrations of Triptolide (Selleck, S3604) were added. Organoid growth was monitored and recorded with 10 × images captured. At the experiment’s endpoint, Calcein-AM was added to stain viable organoids, and fluorescence images were taken after 30 min of staining. Organoid viability was further assessed using an ATP activity assay kit (AiMingMED, 100–347), with luminescence readings reflecting the total viability of all organoids in each well.

### Lactate assay

Cell were collected and resuspended in lactate assay buffer. The suspension was quickly pipetted up and down, then centrifuged at maximum speed at 4 °C for 2–5 min to remove insoluble material. The supernatant was collected and kept on ice for further analysis. The remaining steps were performed following the manufacturer’s protocol (Abcam, ab65331). Absorbance at 450 nm was recorded, and lactate concentrations were calculated based on the standard curve.

### Exosome isolation and characterization

SKOV3 cells were cultured in complete medium supplemented with exosome-depleted FBS (Umibio, UR50202) for 24 h. The collected supernatant was centrifuged at 2000 × g for 30 min to remove cells and debris. The supernatant was then mixed with reagents from the Total Exosome Isolation Kit (Invitrogen, 4478359) and incubated overnight. Exosomes were collected the next day after centrifugation.

For electron microscopy, extracted exosomes were applied onto copper grids, followed by staining with uranyl acetate for 1 min, and the grid was air-dried at RT for a few minutes. Imaging was performed using a transmission electron microscope at 100 kV. For Nanoparticle Tracking Analysis (NTA), exosomes were analyzed for particle size and concentration using a NanoFCM system. Western blot was performed using antibodies against exosome markers *CD9* (Proteintech, 20597-1-AP), *CD81* (Proteintech, 66866-1-Ig), and *TSG101* (Proteintech, 28283-1-AP) to confirm the presence of exosomes.

### Nuclear and cytoplasmic protein extraction and western blot

Cells were lysed and centrifuged at 10,000 × g for 10 min at 4 °C, and the supernatant was collected. Nuclear and cytoplasmic proteins were extracted using a commercial kit (Beyotime, P0028) to ensure efficient separation. Equal amounts of protein were separated by SDS-PAGE and transferred onto PVDF membranes. Membranes were blocked with quick-blocking solution (Beyotime, P0252) and incubated with primary antibodies including *ITGA5* (Proteintech, 10569-1-AP), *ITGB1* (Proteintech, 26918-1-AP), *β-actin* (Proteintech, 66009-1-Ig), *Slug* (Proteintech, 12129-1-AP), *N-cadherin* (Proteintech, 22018-1-AP), *LDHA* (Proteintech, 21799-1-AP), and *YAP1* (Proteintech, 13584-1-AP). Membranes were incubated with HRP-secondary antibodies the following day and visualized. For organoid total protein extraction, the procedure was similar, with lysis performed for 30 min and pipetting every 5 min to ensure thorough disruption.

### Animal models

OC tissues obtained from surgical patients were cut into small pieces (2–3 mm^3^), with necrotic and non-tumor parts removed. The prepared tumor tissues were subcutaneously implanted into the right posterior axilla of 4–6 week-old female NSG mice (n = 3). Tumor growth was regularly monitored, and after two stable passages in NSG mice, the tumors were harvested and re-implanted into new mice for treatment with sodium oxamate and stiripentol. Tumor size was measured, and mice were randomly assigned to treatment groups and a control group. The treatment group received sodium oxamate (500 mg/kg) and stiripentol (200 mg/kg). Tumor growth was monitored and recorded, with volume calculated using the formula V = (L × W^2^) / 2. After two weeks, and tumors were extracted and weighed.

To establish the SKOV3 cell-derived subcutaneous xenograft model, SKOV3 sgCtrl and sgPPP2CA cells were implanted subcutaneously into 4–6 week-old female athymic nude mice (n = 5). Each mouse received 5 × 10⁶ cells mixed with matrigel in a 1:1 ratio. Tumor growth was recorded as previously described. The mice were closely observed for any health changes, and at the experimental endpoint, tumors were excised and weighed. Tumor samples were harvested for immunofluorescence, immunohistochemistry, and HE staining. A similar procedure was followed for shCtrl and shITGA5/ITGB1 cell groups.

To further investigate the effect of Triptolide on *PPP2CA*-knock out tumors, 20 nude mice were randomly divided into two groups (n = 10) and subcutaneously implanted with sgCtrl and sgPPP2CA cells, Following implantation, each group was further divided into a control group and a Triptolide treatment group (0.3 mg/kg), Triptolide was administered via intraperitoneal injection every other day for two weeks. At the experimental endpoint, mice were euthanized and tumors were excised.

### Co-culture experiment

For the indirect co-culture experiment, mesothelial cells were seeded at 2 × 10^5^ cells/2.6 mL in a six-well plate. Different cells were seeded at 1 × 10^5^ cells/1.5 mL per well into Millicell cell culture inserts (Millipore, PTHT06H48) with 0.4 μm pore size. After 48 h of co-culture, HPMC were counted and resuspended for transwell migration assay. The number of migrating cells was recorded and representative images were captured after 24 h, as previously described.

For the direct co-culture with exosomes, mesothelial cells were adjusted to 2 × 10^5^ cells/mL in serum-free medium. Then, 3 mg of exosomes were mixed with 200 μL of the mesothelial cell suspension and added to an transwell insert. The number of migrated cells was recorded after 24 h, and representative images were captured.

### Tissue and organoid immunofluorescence

Tumor tissue sections from mice were subjected to antigen retrieval to expose the epitopes. Subsequently, 3% BSA was applied to block nonspecific binding sites at RT for 30 min, after which the primary antibody *YAP1* (Proteintech, 13,584–1-AP) was added. Sections were incubated with a fluorescently labeled secondary antibody at RT, followed by nuclear staining with DAPI. *YAP* expression and localization were assessed.

For organoid immunofluorescence, the process mirrors the above steps. After incubation with the secondary antibody, Tyramide signal amplification (TSA) staining was applied using a fluorophore-labeled tyramide reagent. Once TSA staining was complete, blocking was performed before introducing new antibodies for subsequent labeling.

### Hematoxylin–eosin (HE) and immunohistochemistry staining

For HE staining, tissue sections were deparaffinized in xylene and rehydrated through a graded ethanol series. After rehydration, sections were stained in hematoxylin for 5 min to visualize nuclei. The sections were then rinsed under running water and differentiated in acidic alcohol. Subsequently, they were counterstained with eosin for approximately 30 s to stain the cytoplasm and extracellular matrix. The sections were dehydrated in ethanol, cleared with xylene, then prepared for microscopic examination.

For immunohistochemistry, staining, tissue sections were incubated with primary antibodies against *ITGA5*, *ITGB1*, and *Ki67*. DAB was used for color development, with staining monitored microscopically. After rinsing with water to remove excess DAB, the sections were counterstained with hematoxylin for 30 s, dehydrated, and mounted.

### RNA sequencing and data analysis

RNA from tumor cells was extracted using the Rneasy Mini Kit (QIAGEN, 74104), RNA concentration and quality were assessed respectively. Library preparation was performed for paired-end multiplexed sequencing on the Illumina platform. Sequencing was carried out on the Illumina Novaseq 6000, generating 150 bp paired-end reads. Differentially expressed genes were identified based on a p-value < 0.05 using the limma package. Raw sequence data are accessible in the Genome Sequence Archive (GSA: CRA015120) via the National Genomics Data Center at https://ngdc.cncb.ac.cn/gsa. Gene set annotation was sourced from MsigDB, and pathway enrichment was performed using the fgsea package (version 1.30.0).

### Survival analysis

Glycolysis scores were first calculated for each patient in the TCGA-OV dataset by aggregating the expression values of genes included in the KEGG “Glycolysis / Gluconeogenesis” pathway (hsa00010), as defined in the Molecular Signatures Database (MSigDB; https://www.gsea-msigdb.org/gsea/msigdb/collections.jsp). The full list of genes used for scoring is provided in Supplementary File 1. This was performed using the gsva function from the GSVA R package, with the parameters set to method = ”gsva” and kcdf = ”Gaussian”. Based on these glycolysis scores, the surv_cutpoint function from the survminer package was used to determine the optimal cutoff value for overall survival. Patients with scores above this cutoff were classified into the “high glycolysis” group, and those below into the “low glycolysis” group. Kaplan–Meier survival analysis and the Log-rank test were then used to compare survival differences between the two groups.

### Statistical analysis and data availability

All statistical analyses were conducted using GraphPad Prism 9. For comparisons between two groups, Student’s t-test was used. A p-value of less than 0.05 was considered statistically significant. Data are presented as mean ± SEM. RNA expression data for OC cell lines are available from the Broad Institute’s DepMap portal (DepMap Cancer Cell Line Encyclopedia (CCLE) Public 23Q2; https://depmap.org/portal/download/). Public RNA sequencing datasets of metastatic OC patients (GSE218939, GSE137237 and GSE98281) are available at GEO database.

## Results

### PPP2CA is a regulator of metabolic homeostasis in OC

**Fig. 1:**
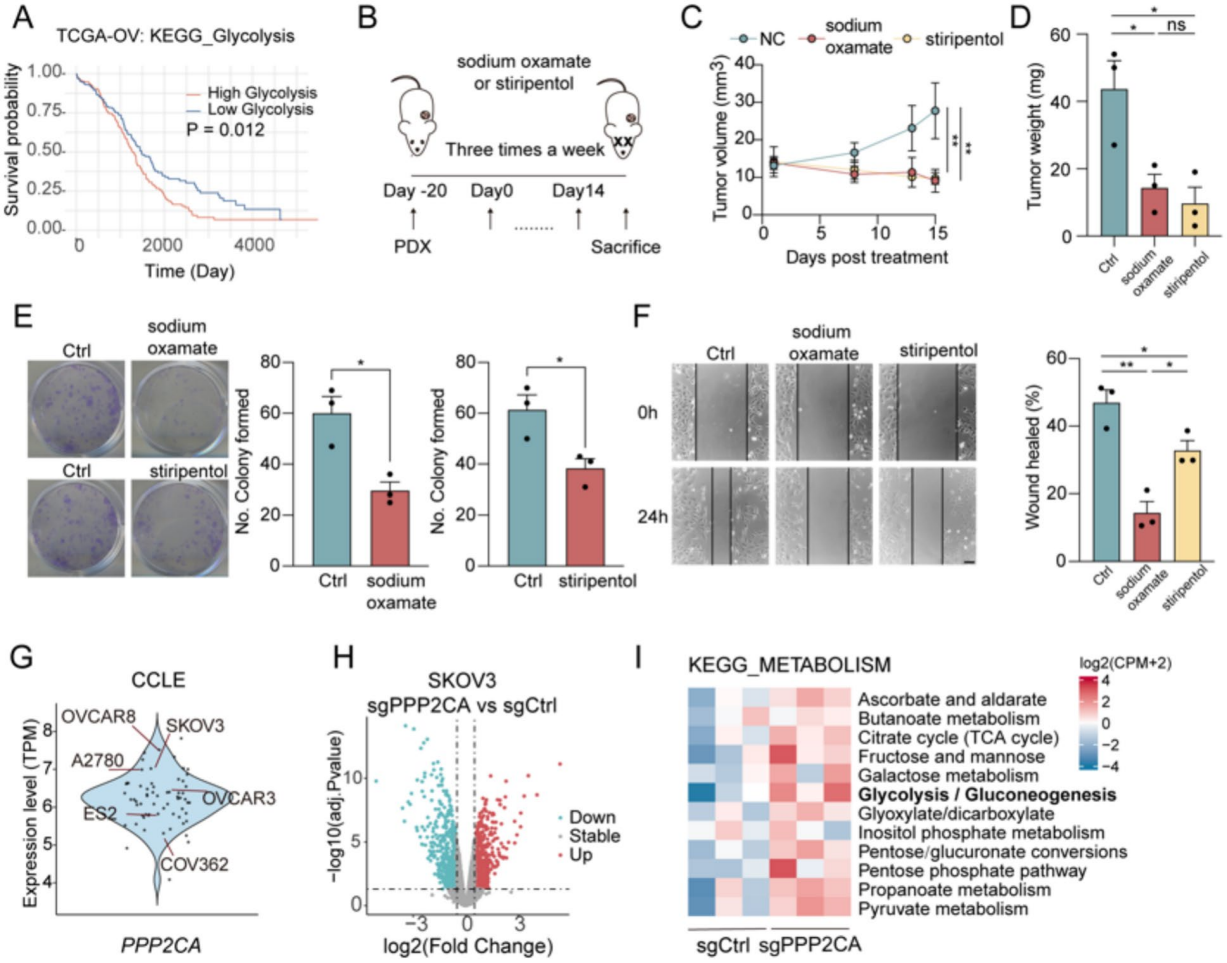
Antilactate agents effectively suppress OC, while *PPP2CA* knockout induces elevated lactate levels in OC Cells. **A** Kaplan–Meier survival analysis of the TCGA-OV cohort (n = 373) comparing overall survival between OC patients with high and low glycolytic gene expression (HR = 1.406, 95% CI 1.078–1.834, p = 0.012). **B** Schematic of the PDX model. OC patient-derived cells were transplanted into NSG mice and passaged twice for stabilization. Treatment with sodium oxamate or stiripentol was administered three times per week from Day 0 to Day 15, followed by tumor excision and analysis. **C**, **D** Measurement of PDX tumor volume (**C**) and tumor weight (**D**) at the experimental endpoint. **E**, **F** Colony formation (**E**) and wound healing (**F**) assays in SKOV3 cells treated with sodium oxamate or stiripentol. **G** Scatter plot showing the expression of *PPP2CA* in different OC cell lines from the CCLE database. **H**, **I** RNA sequencing analysis of *PPP2CA*-knockout and control cells, including volcano plot of differentially expressed genes (**H**) and KEGG pathway enrichment analysis (**I**). *ns* not significant; *p < 0.05, **p < 0.01. Scale bar = 50 µm

**Fig. 2:**
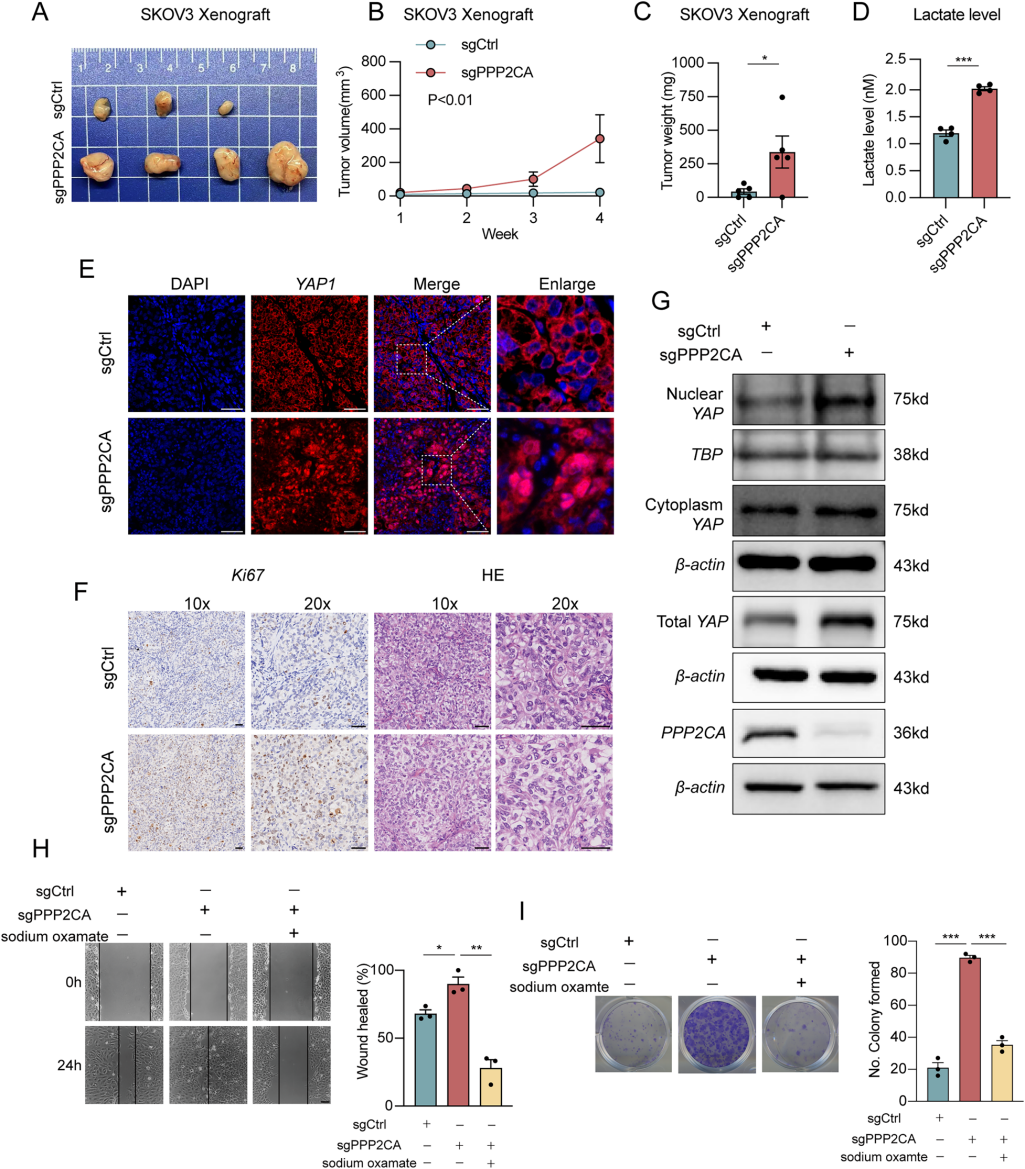
*PPP2CA* deficiency drives OC progression via lactate production and *YAP* nuclear translocation. **A**–**C** SKOV3 xenograft model established by subcutaneous injection of control and *PPP2CA*-knockout cells in nude mice. Representative images were taken (**A**) and tumor volume was measured over a 4-week period (**B**), and tumor weight was recorded at the experimental endpoint (**C**). **D** Measurement of lactate levels in *PPP2CA*-knockout cells and control cells. **E** Immunofluorescence staining of *YAP1* localization in xenograft tumor tissues from *PPP2CA*-knockout and control groups. **F** HE and immunohistochemistry staining of xenograft tumors derived from control and *PPP2CA*-knockout tumors. **G** Western blot of nuclear and cytoplasmic fractions of *YAP1* in control and *PPP2CA*-knockout cells. **H** Wound healing assay in *PPP2CA*-knockout cells with/without sodium oxamate treatment. Representative images at 0 h and 24 h and quantification of wound closure. **I** Colony formation assay in *PPP2CA*-knockout with/without sodium oxamate treatment. Representative images of colony formation and quantification of colony numbers. *p < 0.05, **p < 0.01, ***p < 0.001. Scale bar = 50 µm

Glycolysis is a metabolic pathway that degrades glucose to produce ATP. In anaerobic conditions or within tumors, pyruvate is predominantly converted to lactate instead of entering the mitochondria for oxidative phosphorylation, with lactate production marking the endpoint of anaerobic glycolysis, known as the *Warburg effect* [30]. Given the critical role of glycolysis in cancer, we first examined its association with OC prognosis and found that elevated glycolytic activity correlated with poorer outcomes (Fig. 1A). To further investigate the role of lactate in OC, we focused on *LDHA*, a key enzyme in lactate production, which can be inhibited by sodium oxamate. Additionally, stiripentol, an antiepileptic drug, has been shown to impair lactate production in gastric cancer, exhibiting antitumor effects [31]. Therefore, we treated OC cells with sodium oxamate or stiripentol and observed significant inhibition of proliferation and migration, as evidenced by reduced colony formation and impaired wound healing (Fig. 1E, F). To further validate the role of lactate in OC progression and assess the therapeutic potential of sodium oxamate and stiripentol in a more clinically relevant setting, we established an OC patient-derived xenograft (PDX) model, demonstrating that sodium oxamate or stiripentol markedly reduced tumor growth (Fig. 1B–D), emphasizing the role of lactate accumulation in tumor progression. Despite confirming lactate’s involvement in OC progression, its upstream regulatory mechanisms remained unclear. *PPP2CA* has been recognized as a tumor suppressor involved in metabolic regulation, but its role in lactate metabolism in OC had yet to be determined. To address this, we first analyzed *PPP2CA* expression across OC cell lines using the CCLE and identified SKOV3 cells as having high *PPP2CA* expression (Fig. 1G), We then generated *PPP2CA*-knockout cells using CRISPR-Cas9 gene editing and confirmed via western blot and Sanger sequencing (Fig. 2J and S1E). To explore the impact of *PPP2CA* on metabolic homeostasis, we measured lactate levels in *PPP2CA*-knockout cells and found a significant increase (Fig. 2D). Functionally, *PPP2CA* knockout significantly enhanced OC cell proliferation and migration, as evidenced by accelerated wound healing (Fig. 2H), and enhanced colony formation (Fig. 2I). Notably, these effects were largely reversed when *PPP2CA*-knockout cells were treated with sodium oxamate (Fig. 2H, I), confirming that *PPP2CA* loss promotes lactate-driven OC progression. To further validate the role of lactate, we treated OC cells with exogenous L-lactate and observed significantly enhanced migration in wound healing assay (Fig S1A), providing additional evidence that lactate directly promotes OC cell motility. To elucidate the molecular mechanisms underlying *PPP2CA*’s role in OC, we performed RNA sequencing on *PPP2CA*-knockout cells and found significant enrichment of glycolysis/gluconeogenesis KEGG pathways (Fig. 1H–I), underscoring *PPP2CA*’s pivotal role in metabolic homeostasis.

### PPP2CA deficiency drives OC progression via lactate production and YAP nuclear translocation

To further investigate *PPP2CA*’s role in tumorigenesis, we implanted *PPP2CA*-knockout and control cells subcutaneously in mice to assess their tumorigenic potential in vivo. *PPP2CA* deficiency accelerated tumor growth, with larger tumor volumes and greater tumor mass observed in the *PPP2CA*-knockout group compared to controls (Fig. 2A–C). Histological examination of the tumors revealed more prominent nuclear atypia and the presence of vacuolar structures in the cytoplasm following *PPP2CA* knockout (Fig. 2F). Additionally, Immunohistochemical staining for *Ki-67* further confirmed increased proliferation in the *PPP2CA*-knockout tumors (Fig. 2F), supporting its role as a tumor suppressor in OC progression.

*PP2A* regulates the Hippo pathway by dephosphorylating *YAP*, balancing its activation. Normally, *MST1/2* and *LATS1/2* kinases phosphorylate *YAP*, restricting it to the cytoplasm. After dephosphorylation by *PP2A*, *YAP* translocates to the nucleus, activating genes linked to proliferation and tumor growth [32, 33]. Given this, we hypothesized that *PPP2CA* deficiency may promote *YAP* nuclear translocation. To test this, we performed immunofluorescence analysis on *PPP2CA*-knockout tumors, which showed a significant increase in nuclear *YAP* localization, while in control tumors, *YAP* was predominantly confined to the cytoplasm (Fig. 2E). To further quantify *YAP* subcellular distribution, we fractionated cytoplasmic and nuclear proteins and found that knock-out of *PPP2CA* exhibited a significant increase in nuclear *YAP* levels, with no apparent change in cytoplasmic *YAP* levels (Fig. 2G).

### PPP2CA dysregulation mediates ITGA5/ITGB1 upregulation, driving OC progression

Gene Set Enrichment Analysis (GSEA) revealed significant enrichment of integrin interactions and collagen fibril assembly pathways in *PPP2CA*-knockout cells compared to the control group (Fig S1C). Additionally, GO analysis identified pathways related to EMT, ERBB2, and Ras signaling (Fig S1D) suggesting that *PPP2CA* loss may drive EMT and activate integrin-mediated signaling. Given that the acidic TME and elevated lactate levels can alter the ECM composition, and considering the critical role of integrins in cell-ECM interactions [34], we conducted further analysis to identify integrin family members linked to OC progression to identify potential key regulators.

**Fig. 3:**
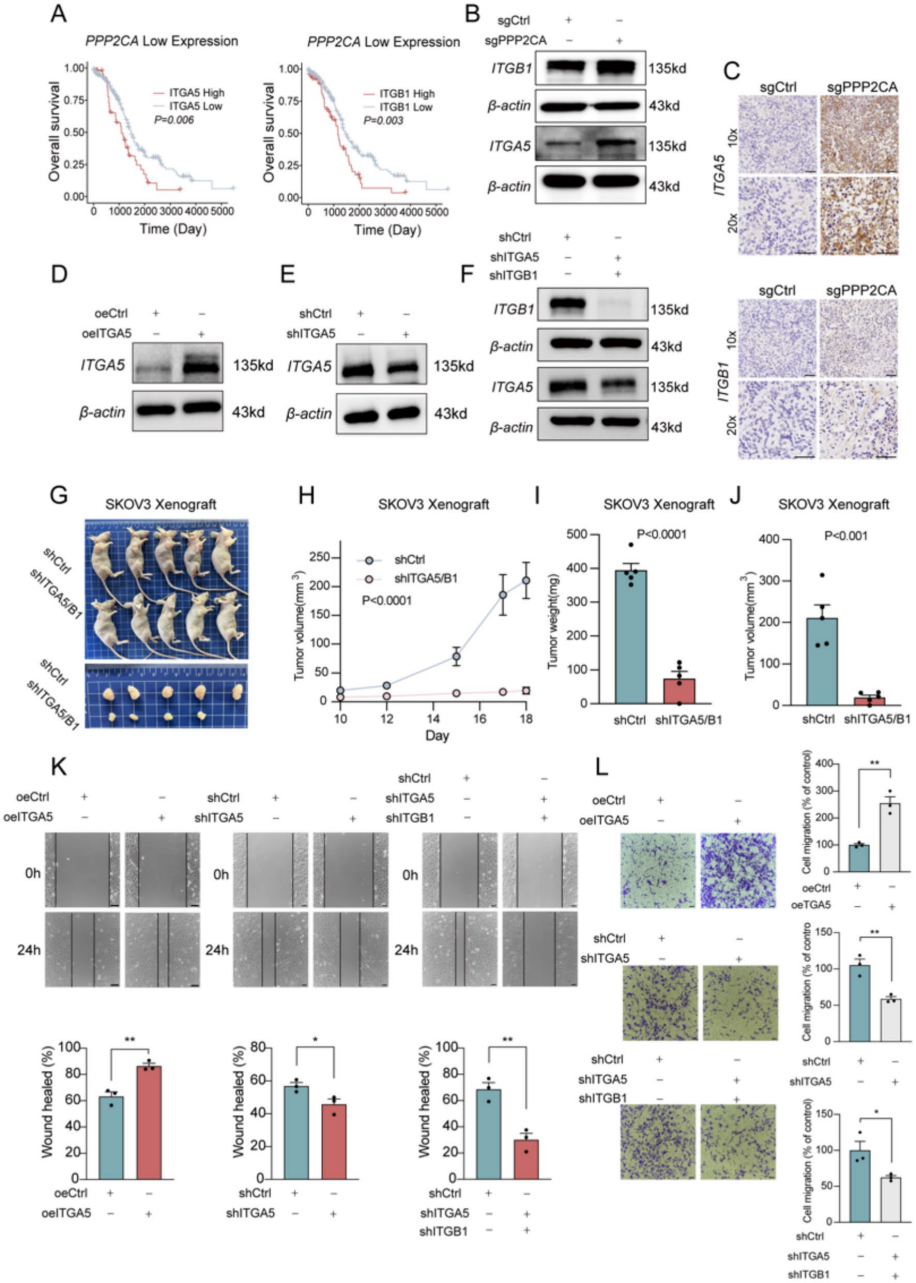
*PPP2CA* dysregulation mediates *ITGA5/ITGB1* upregulation, driving OC progression. **A** Kaplan–Meier survival curves for OC patients with low *PPP2CA* expression. **B** Western blot analysis showing *ITGA5* and *ITGB1* protein levels in control and *PPP2CA*-knockout cells. **C** Immunohistochemistry staining of *ITGA5* and *ITGB1* in tumors derived from control and *PPP2CA*-knockout tumors. **D**–**F** Verification of the efficiency of *ITGA5* overexpression (**D**), knockdown (**E**), and *ITGA5/ITGB1* double knockdown (**F**). **G**–**J** Tumor growth assessment in SKOV3 xenografts with *ITGA5* and *ITGB1* knockdown compared to controls. Representative images of tumors (**G**), tumor growth curves (**H**), tumor weight (**I**) and volume (**J**) at the experimental endpoint. **K** Wound healing and transwell migration assays (**L**) were performed on SKOV3 cells with *ITGA5* overexpression (oeITGA5), *ITGA5* knockdown (shITGA5), or *ITGA5/ITGB1* double knockdown (shITGA5/shITGB1) to assess their effects on cell migration. *ns* not significant; *p < 0.05, **p < 0.01. Scale bar = 50 µm

To assess the expression patterns of integrins in OC metastases, we analyzed three OC datasets from the NCBI GEO database. *ITGB1* was identified as the most highly expressed integrin in 2 out of 3 datasets (Fig S2A). Since integrins typically function as heterodimers, *ITGA5* and *ITGB1* combine to form the *α5β1* complex, a key ECM receptor that regulates cell adhesion, migration, and mechanotransduction. Correlation analysis confirmed a significant co-expression of *ITGA5* and *ITGB1* in OC tissues (Fig S2B). In patients with low *PPP2CA* expression, elevated *ITGA5* and *ITGB1* levels were strongly linked to poorer survival outcomes, with high expression of either integrin subunit alone also correlating with reduced overall survival (Fig. 3A, Fig S2E). However, in patients with high *PPP2CA* expression, neither *ITGA5* nor *ITGB1* exhibited a significant association with overall survival, suggesting that *PPP2CA* may play a regulatory role in integrin-mediated tumor progression (Fig S2D).

Given this potential regulatory relationship, we next investigated how *PPP2CA* loss impacts *ITGA5* and *ITGB1* expression. We found that *PPP2CA*-knockout tumors revealed an upregulation of *ITGA5* and *ITGB1* (Fig. 3C), and this finding was further validated by western blot analysis at the protein level. These results demonstrated a significant increase in both *ITGA5* and *ITGB1* expression in *PPP2CA*-knockout cells (Fig. 3B). To further investigate the mechanism underlying *ITGA5/ITGB1* upregulation, we examined the potential involvement of *YAP1*, a key transcriptional regulator activated following *PPP2CA* loss. Mechanistically, silencing *YAP1* in *PPP2CA*-deficient cells markedly decreased *ITGA5* and *ITGB1* expression (Fig S1B). In contrast, knockdown of *ITGA5*/*ITGB1* had no impact on *YAP1* levels (Fig S1F), suggesting a unidirectional regulatory axis from *PPP2CA* to *YAP1* to *ITGA5/ITGB1*. In addition, to validate the functional contribution of *ITGA5/ITGB1* to the enhanced migratory phenotype driven by *PPP2CA* loss, we further generated double knockdown models in the *PPP2CA*-deficient cells. Compared to *PPP2CA*-knockout cells, simultaneous knockdown of *ITGA5/ITGB1* markedly reduced cell migration in wound healing assay (Fig S1G, H), supporting their essential role as downstream mediators in promoting OC cell motility following *PPP2CA* depletion. To explore their functional relevance, we generated *ITGA5/ITGB1* double knockdown cell lines (Fig. 3F) and assessed their impact on tumor growth in vivo. The results showed a significant reduction in tumor growth in the *ITGA5/ITGB1* double-knockdown group (Fig. 3G–J), indicating that these integrins contribute to OC progression.

### ITGA5 drives OC cell migration

To further delineate the individual functions of *ITGA5* and *ITGB1* in OC metastasis, we established *ITGA5* and *ITGB1* single knockdown cell lines (Fig. 3E and Fig S2E). Given that integrins primarily mediate cell–matrix interactions as heterodimers, this aimed to determine whether the pro-migratory effects of the α5β1 complex are predominantly driven by *ITGA5*, *ITGB1*, or a synergistic interaction between the two. Transwell migration and wound healing assays demonstrated that both *ITGA5* knockdown and *ITGA5/ITGB1* double knockdown significantly inhibited cell migration (Fig. 3K, L). Notably, dual knockdown resulted in a stronger inhibitory effect in the wound healing assay than *ITGA5* knockdown alone (Fig. 3K,L), whereas *ITGB1* knockdown had no significant impact on migration (Fig S2F, G). These findings suggest that *ITGA5*, rather than *ITGB1*, is the dominant regulator of migration in the *α5β1* complex. To further confirm this gain-of-function effect, we generated *ITGA5*-overexpressing cells (Fig. 3D) and assessed their migratory capacity (Fig. 3K, L). The results showed that *ITGA5* overexpression significantly enhanced migration, reinforcing its role as a key pro-metastatic factor.

### Exosomal ITGA5 drives tumor cell preference for metastasis toward HPMCs

**Fig. 4:**
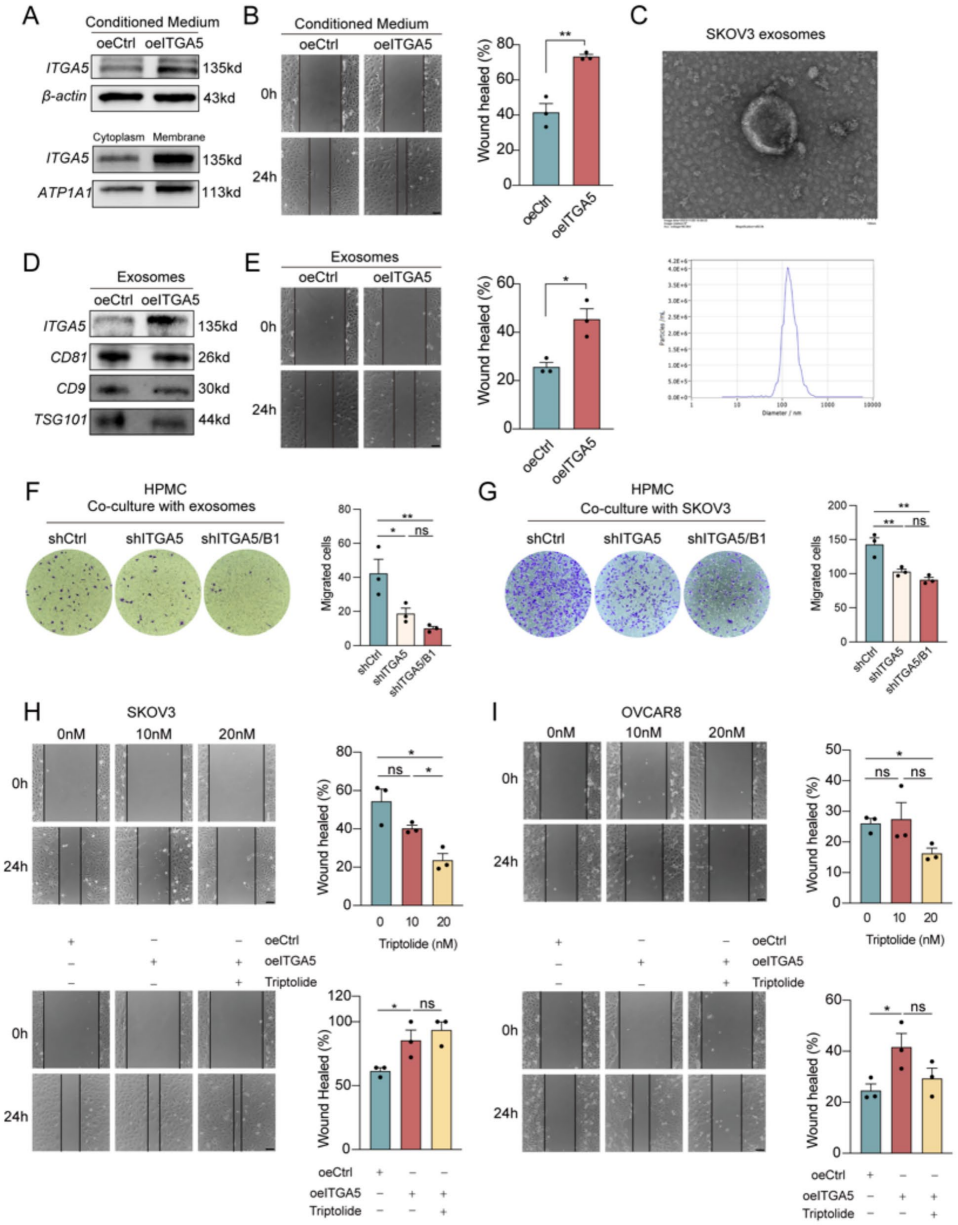
Exosomal transfer of *ITGA5* promotes tumor cell preference for metastasis toward HPMCs. **A** Western blot analysis of *ITGA5* expression in SKOV3 cells after 48 h treatment with conditioned medium from *ITGA5*-overexpressing cells. **B** Wound healing assay of SKOV3 cells cultured in conditioned medium from *ITGA5*-overexpressing (oeITGA5) cells or control medium. **C** Electron microscopy images of exosomes derived from *ITGA5*-overexpressing cells, nanoparticle tracking analysis showing the size distribution of the exosomes. **D** Western blot analysis of exosomes isolated from control and *ITGA5*-overexpressing cells, detecting exosome markers (CD81, CD9, TSG101) and *ITGA5* expression. **E** Wound healing assay of SKOV3 cells treated with exosomes isolated from *ITGA5*-overexpressing cells or control cells. **F**, **G** Migration assay of HPMCs treated with exosomes isolated from various cell lines (**F**) or co-cultured with cells with *ITGA5* knockdown or *ITGA5/ITGB1* double knockdown (**G**). **H, I** Wound healing assays were performed on SKOV3 and OVCAR8 cells treated with Triptolide at different concentrations for 24 h, as well as on SKOV3 and OVCAR8 cells overexpressing *ITGA5*, following Triptolide treatment. *ns* not significant; *p < 0.05, **p < 0.01. Scale bar = 50 µm

Since *ITGA5* promotes OC cell migration, we next investigated its role in intercellular communication and potential impact on metastasis. Integrins are commonly incorporated into exosomes, which can either interact with target cell surface receptors or be internalized, subsequently modulating recipient cell behavior and influencing metastatic potential [35, 36]. Moreover, exosomal integrins facilitate the formation of pre-metastatic niches, directing cancer cells to specific metastatic sites [37]. To assess whether *ITGA5*-expressing cells influence the migratory behavior of neighboring OC cells, we utilized overexpressing *ITGA5* cell lines, confirming correct membrane localization of *ITGA5* through membrane fractionation assays (Fig. 4A). Conditioned medium from *ITGA5*-overexpressing cells was found to enhance the migratory ability of parental OC cells (Fig. 4A, B), suggesting the involvement of secreted factors in *ITGA5*-mediated intercellular communication. Given that integrins are frequently transported via exosomes, we hypothesized that exosomal *ITGA5* might contribute to this effect. To test this, exosomes were isolated from *ITGA5*-overexpressing cells, characterized via electron microscopy and nanoparticle size analysis (Fig. 4C), and validated by western blot for exosomal markers and *ITGA5* expression (Fig. 4D). Migration assays demonstrated that exosomal *ITGA5* significantly enhanced OC cell motility, supporting the notion that exosomes serve as a vehicle for *ITGA5*-mediated intercellular signaling (Fig. 4E).

To further explore whether *ITGA5* reaches peritoneal mesothelial cells via exosomes and influences their behavior, we isolated and characterized normal HPMCs (Fig S3A). We then examined the role of exosomal *ITGA5* in modulating HPMC migration. Co-culture experiments with *ITGA5*-knockdown tumor cells (shITGA5, shITGA5/ITGB1) in a non-contact system or treatment with exosomes derived from these cells showed a significant reduction in HPMC migration (Fig. 4F, G). The consistent decrease in migration under both conditions suggests that exosomal *ITGA5* contributes to regulating HPMC motility. Furthermore, fluorescence imaging confirmed successful internalization of tumor-derived exosomes by HPMCs (Fig S3B), providing direct evidence of exosomal uptake. These findings indicate that *ITGA5*-containing exosomes influence HPMC behavior, suggesting a potential role in shaping the peritoneal microenvironment.

### Triptolide targets ITGA5 to reverse PPP2CA knockout-induced tumorigenesis in OC

**Fig. 5:**
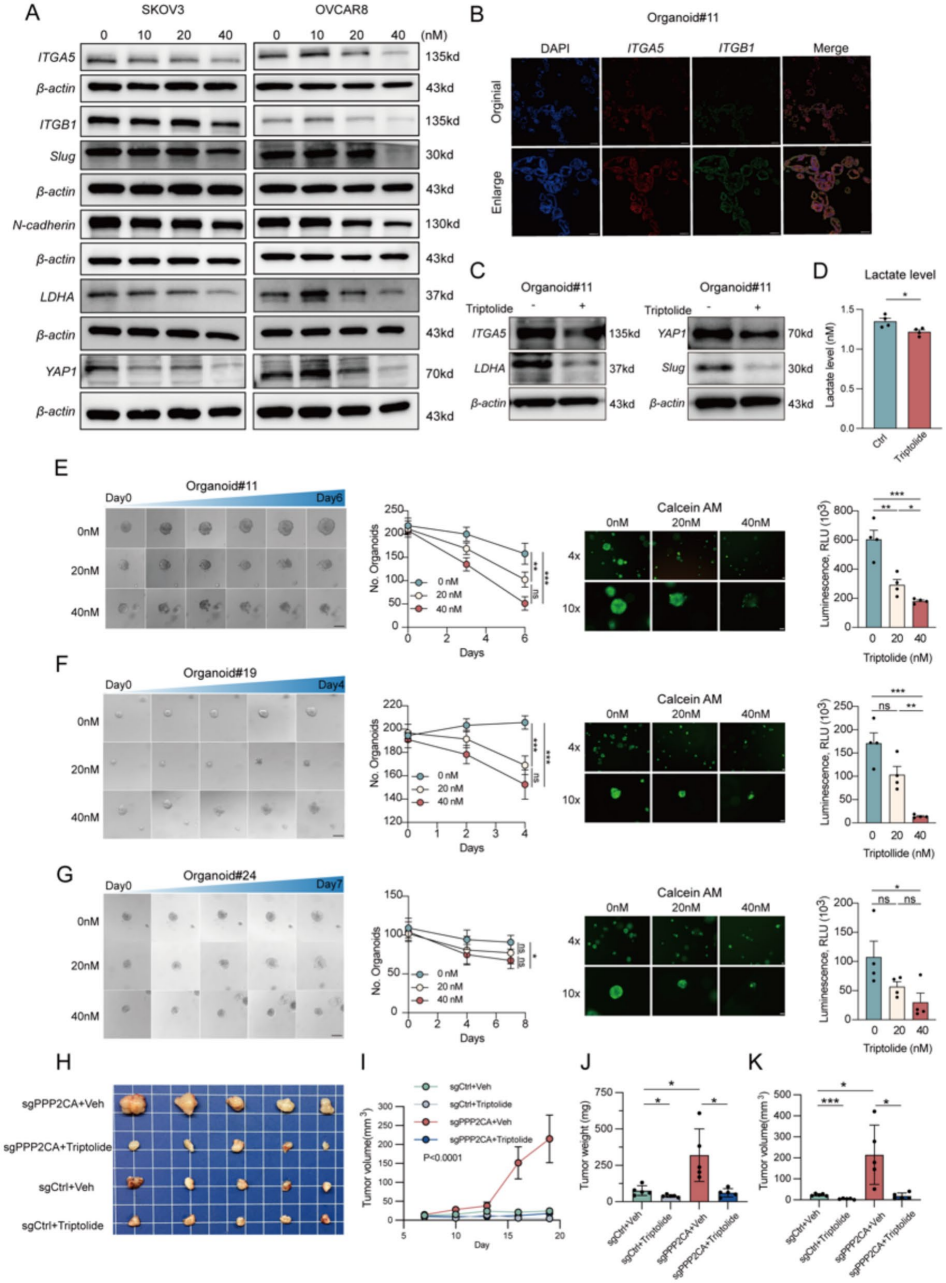
Triptolide exhibits efficacy against patient-derived OC organoids. **A** Western blot analysis of SKOV3 and OVCAR8 cells treated with Triptolide at different concentrations. **B** Immunofluorescence of Triptolide-treated OC organoids (#11) stained for DAPI (blue), *ITGA5* (red), and *ITGB1* (green). Scale bar = 100 µm. **C** Western blot analysis of Triptolide-treated organoids. **D** Lactate levels in Triptolide-treated OC cells. **E**–**G** Representative images and viability analysis of Triptolide-treated OC organoids. Calcein-AM staining (4 × and 10 × magnification) and luminescence assay assess viability. **H**–**K** Tumor growth assessment in *PPP2CA*-knockout xenografts. Representative images of tumors (**H**), tumor growth curves (**I**), tumor weight (**J**) and volume (**K**) at the experimental endpoint. *ns* not significant; *p < 0.05, **p < 0.01, ***p < 0.001. Scale bar = 50 µm

Triptolide has been previously reported to exhibit therapeutic efficacy in OC cell lines [38], However, its precise mechanism in modulating tumor migration remains unclear. To determine whether Triptolide interferes with *ITGA5*-mediated metastatic progression, we first evaluated its effects on OC cell motility. Transwell and wound healing assays demonstrated that Triptolide treatment significantly reduced the migration ability of SKOV3 and OVCAR8 cells. Since we had already established *ITGA5*-overexpressing SKOV3 cells, we further overexpressed *ITGA5* in OVCAR8 cells to confirm whether Triptolide-mediated suppression of migration was dependent on *ITGA5* (Fig S2C). In both cell lines, *ITGA5* overexpression attenuated Triptolide-induced migration inhibition (Fig. 4H, I). Western blot analysis further confirmed that Triptolide (20 nM and 40 nM) downregulated *ITGA5* and *ITGB1* expression, indicating that Triptolide exerts direct inhibitory effects on the *integrin α5β1* complex (Fig. 5A).

Additionally, the expression of the mesenchymal marker *N-cadherin* and the key EMT transcription factor *Slug* was significantly downregulated at 40 nM Triptolide, suggesting that Triptolide has a marked inhibitory effect on the EMT process (Fig. 5A). To determine whether Triptolide directly affects metabolic alterations associated with the *PPP2CA-ITGA5* axis, we assessed its impact on *LDHA* expression and lactate production. Western blot analysis revealed that Triptolide treatment significantly reduced *LDHA* expression (Fig. 5A), suggesting that Triptolide interferes with lactate metabolism in OC cells. Lactate quantification assays further confirmed this effect, showing that Triptolide treatment significantly decreased lactate levels (Fig. 5D). To further evaluate Triptolide’s therapeutic potential, we established subcutaneous xenograft models using *PPP2CA*-knockout and control OC cells in nude mice and administered Triptolide treatment. Triptolide significantly suppressed tumor growth in the *PPP2CA*-knockout group within two weeks, effectively reversing the tumor-promoting effects of *PPP2CA* deficiency (Fig. 5H–K). However, its inhibitory effect in the control group was less pronounced, this discrepancy may stem from intrinsic differences in tumor growth rates—slower-growing control tumors may have limited the extent of further inhibition, whereas *PPP2CA*-knockout tumors, characterized by *ITGA5* overexpression and lactate accumulation, likely created a microenvironment more responsive to Triptolide. Furthermore, western blot results showed that *YAP1* expression was reduced following Triptolide treatment (Fig. 5A). These findings suggest that Triptolide may correct the metabolic imbalance induced by *PPP2CA*-knockout, thereby inhibiting tumor progression.

Given our findings that OC-derived exosomal *ITGA5* enhances the migratory capacity of HPMCs, to further evaluate the therapeutic potential of Triptolide in a more clinically relevant setting, we next investigated its anti-metastatic efficacy in a peritoneal metastasis model. Leveraging the homing properties of HPMC-derived exosomes, we first isolated exosomes from HPMCs and subsequently prepared Triptolide-loaded exosomes (Triptolide-Exos) using an ultrasound-assisted loading technique, which allowed efficient drug encapsulation while maintaining exosome structural integrity (Fig S4A). In the ID8 intraperitoneal implantation model using immunocompetent C57BL/6 mice, both free Triptolide and Triptolide-Exos significantly reduced peritoneal tumor burden and metastatic spread. Notably, Triptolide-Exos showed enhanced inhibition of malignant ascites and tumor dissemination, suggesting improved delivery efficiency and local anti-metastatic activity (Fig S4B-E).

### Triptolide shows strong therapeutic potential for personalized treatment in OC organoids

Organoids, as an advanced three-dimensional culture system, are particularly valuable because they retain the cellular heterogeneity inherent to the primary tumor. This system better mimics the in vivo tissue architecture, making it a superior model for studying drug mechanisms and potential therapeutic effects. We established OC organoid models derived from patient tumor tissues (Fig S5A; Table 1). Upon generating stable, passaged organoids, treatment with Triptolide resulted in a marked reduction in organoid numbers, accompanied by significant morphological changes. Specifically, organoids #11 and #19 displayed vacuolation, gradually disintegrating into individual cells or cellular debris post-Triptolide treatment (Fig. 5E, F), while organoid #24 exhibited structural collapse and significant size reduction (Fig. 5G). Further evaluations, including Calcein-AM staining and ATP quantification, demonstrated a substantial reduction in organoid viability following Triptolide treatment, indicating a strong cytotoxic response. Immunofluorescence analysis confirmed the expression of *ITGA5* in the OC organoids, with notable co-localization alongside *ITGB1* (Fig. 5B). Upon collecting organoid proteins treated by Triptolide, we observed a downregulation in the expression of *ITGA5*, as well as *YAP1*, *LDHA*, and *Slug*, which is in accordance with the findings from the cell lines (Fig. 5C). Additionally, immunohistochemistry staining for *Ki-67* showed a significant reduction in proliferative marker staining post-Triptolide treatment (Fig S5B), consistent with diminished cellular proliferation. Collectively, these findings highlight Triptolide’s potent anti-tumor effects in realistic models of OC tumor epithelia.

**Table 1:** Clinical information from patient-derived ovarian cancer organoids

Patient ID	Primary/Relapse	Tumor Site	Pathology	BRCA	ER	PR	Ki67	P53
#11	Primary	Ovary	Endometrioid	/	+	+	30%	WT
#19	Relapse	Colon	HGSOC	mutation	+	–	80%	WT
#24	Primary	Ovary	HGSOC	/	+	–	40%	+ + +

*HGSOC* High-grade serous ovarian cancer

## Discussion

*PP2A* is a pivotal protein phosphatase in the human body and has long been regarded as a key tumor suppressor [39]. Thus, *PP2A* activation emerges as a promising cancer therapy strategy. In this study, we identified its catalytic subunit, *PPP2CA*, plays a critical role in maintaining metabolic homeostasis. When *PPP2CA* becomes inactive or dysfunctional, lactate levels rise, triggering metabolic reprogramming and promoting OC progression. Lactate, beyond its role as a metabolic byproduct, also regulates cell signaling and gene expression through involvement in post-translational histone modifications. Previous research revealed the existence of lactylation, broadening the understanding of lactate’s functions [40]. Subsequent studies showed that lactate not only inactivates *P53* through lactylation [41] but also enhances the immunosuppressive functions of regulatory T cells [42]. Moreover, lactate shields tumor cells from the cytotoxic effects of radiation and chemotherapy [43], demonstrating its multifaceted malignancy within the TME. To validate lactate’s role in OC progression, we used two lactate production inhibitors, sodium oxamate and stiripentol, to inhibit lactate accumulation in our models. These treatments confirmed that lactate accumulation is a key driver of OC progression. However, targeting *LDHA*, the enzyme central to lactate production, could offer a more specific approach to better understanding the exact role of lactate. Furthermore, future studies using immunocompetent animal models will be crucial for exploring how lactate influences the TME, particularly in terms of immune and stromal components.

Our study uncovered *PPP2CA*’s influence on the regulation of integrins and ECM remodeling. Specifically, *Integrin α5* forms a complex with *Integrin β1* and undergoes stress-induced changes, which appear to be driven by the nuclear translocation of *YAP1*. *YAP1* is a central effector of the Hippo signaling pathway, playing a key role in controlling organ size, cell proliferation, and apoptosis [44]. Conventionally, *PP2A* was thought to regulate *YAP* activity through dephosphorylation, yet our findings unexpectedly revealed *YAP*’s nuclear translocation in the context of *PPP2CA* dysregulation. A pan-cancer analysis recently highlighted that *YAP*^on^ tumors—those in which *YAP* suppression is necessary for tumor inhibition, particularly in adherent cell types—show strong dependence on genes related to integrin, ECM, and cytoskeletal regulation [45]. This observation aligns with our findings, where nuclear *YAP* expression contributed to elevated total *YAP* levels in OC cell lines, coinciding with the high expression of *ITGA5*. While we have not yet demonstrated *YAP* lactylation in OC, it is highly plausible that abnormal lactate accumulation affects *ITGA5* expression through *YAP* activation and warrants investigation.

In our study, we show that *ITGA5*, as a downstream target of the *PPP2CA*, plays multiple critical roles in tumor metabolic reprogramming and metastasis. When *PPP2CA* is dysregulated, cellular metabolic balance is disrupted, lactate is produced at an accelerated rate, while *ITGA5* expression is significantly upregulated. *ITGA5* functions intracellularly and is secreted via exosomes, serving as a pro-metastatic signal carrier in the TME. Mesothelial cells, upon receiving these signals, remodel their environment to support tumor expansion and colonization. Triptolide effectively disrupts this process by downregulating *ITGA5*, impairing tumor adhesion and invasion. Additionally, Triptolide suppresses key metabolic enzymes such as *LDHA*, limiting lactate-driven tumor adaptation. Furthermore, we demonstrated Triptolide’s strong therapeutic potential using patient-derived OC organoids, which better preserve tumor heterogeneity and mimic in vivo conditions. Triptolide treatment led to significant suppression of organoid growth, validating its efficacy in a physiologically relevant model. Further supporting these results, in an immunocompetent ID8 peritoneal metastasis model, Triptolide administration markedly reduced peritoneal tumor dissemination and malignant ascites formation, underscoring its potent anti-metastatic effects in vivo.

Our study establishes for the first time that *PPP2CA* dysregulation leads to lactate accumulation, which subsequently enhances *ITGA5* expression. *ITGA5* is secreted via exosomes and participates in signaling between OC cells and HPMCs, promoting a favorable microenvironment for tumor growth. Targeting *ITGA5* with Triptolide significantly inhibits lactate-driven metabolic reprogramming and impedes OC progression, highlighting its potential as a therapeutic approach as a therapeutic approach.

## Supplementary Information

Additional file 1.

Additional file 2.

## Data Availability

Data will be made available on request.

## Abbreviations

OC: Ovarian cancer
PDX: Patient-derived xenograft
TME: Tumor microenvironment
TAMs: Tumor-associated macrophages
PP2A: Protein phosphatase 2A
EMT: Epithelial-mesenchymal transition
OXPHOS: Oxidative phosphorylation
HPMCs: Human peritoneal mesothelial cells
ECM: Extracellular matrix
HSP: Heat shock proteins
ROS: Reactive oxygen species
HE: Hematoxylin–eosin
CCLE: Cancer Cell Line Encyclopedia
GSEA: Gene set enrichment analysis
