# Supplementary material for: Triptolide targets PPP2CA/ITGA5 axis to suppress lactate-driven ovarian cancer progression
Source: Chin Med. 2025 Aug 6;20:122. doi: 10.1186/s13020-025-01174-2 (PMC12326723; doi:10.1186/s13020-025-01174-2)
Supplement: Supplementary file 2 — Additional file 2. [file 13020_2025_1174_MOESM2_ESM.docx]

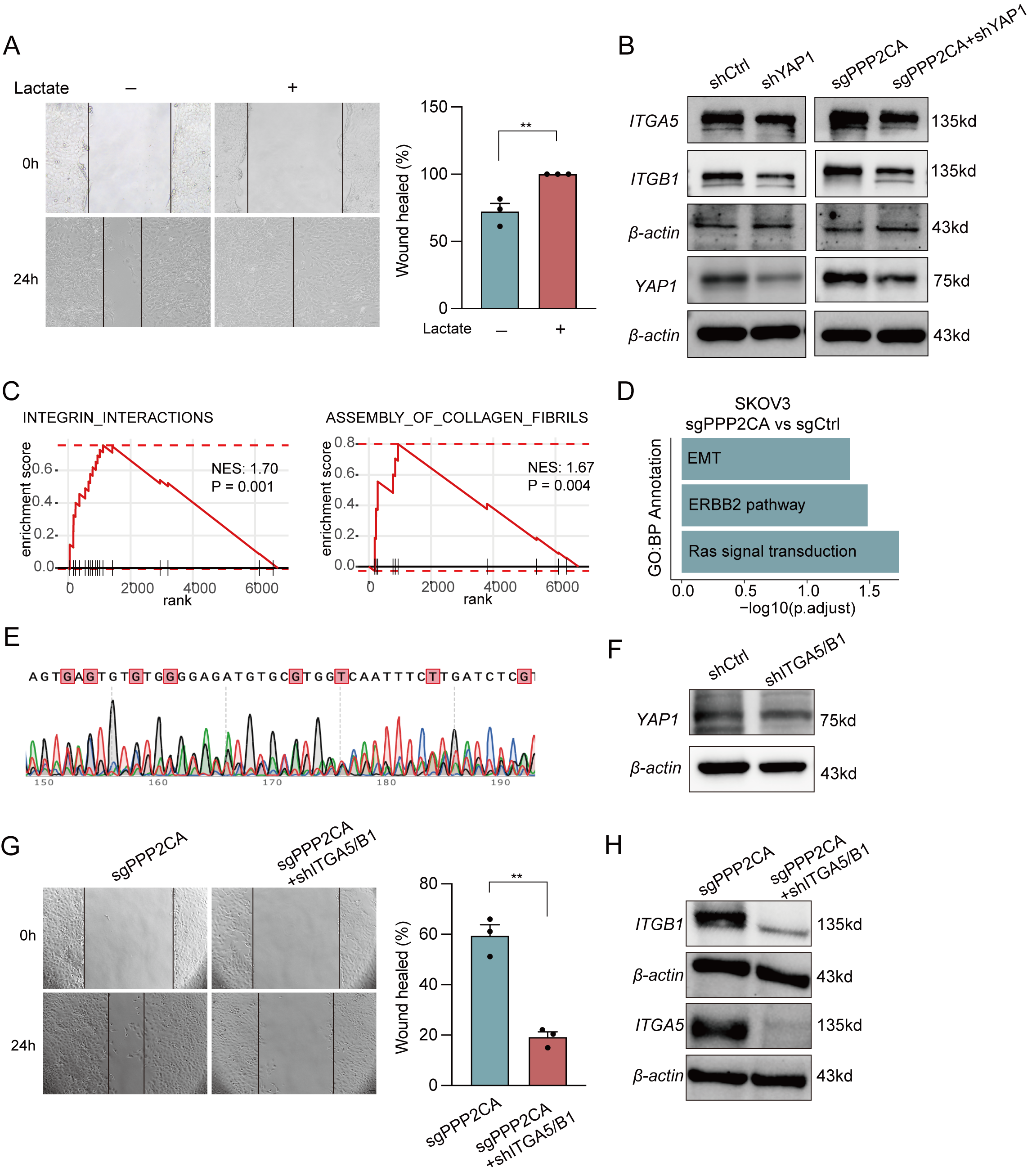


Figure S1. (A) Wound healing assay in SKOV3 cells treated with or without exogenous lactate. (B) Western blot analysis of *ITGA5* and *ITGB1* expression in SKOV3 cells after *YAP1* knockdown, *PPP2CA* knockout, or combined *PPP2CA* knockout and *YAP1* knockdown. (C) GSEA of *PPP2CA*-knock out and control cells. (D) Gene Ontology (GO) and pathway enrichment analysis were performed on RNA sequencing data from *PPP2CA*-knockout and control cells. (E) Sanger sequencing of sgPPP2CA cells to validate *PPP2CA* knockout. (F) Western blot analysis of *YAP1* expression after *ITGA5/ITGB1* knockdown in SKOV3 cells. (G) Wound healing assay in *PPP2CA*-knockout SKOV3 cells with or without *ITGA5/ITGB1* knockdown. (H) Western blot analysis of *ITGA5* and *ITGB1* expression following knockdown in *PPP2CA*-knockout cells. **p < 0.01. Scale bar = 50 µm.


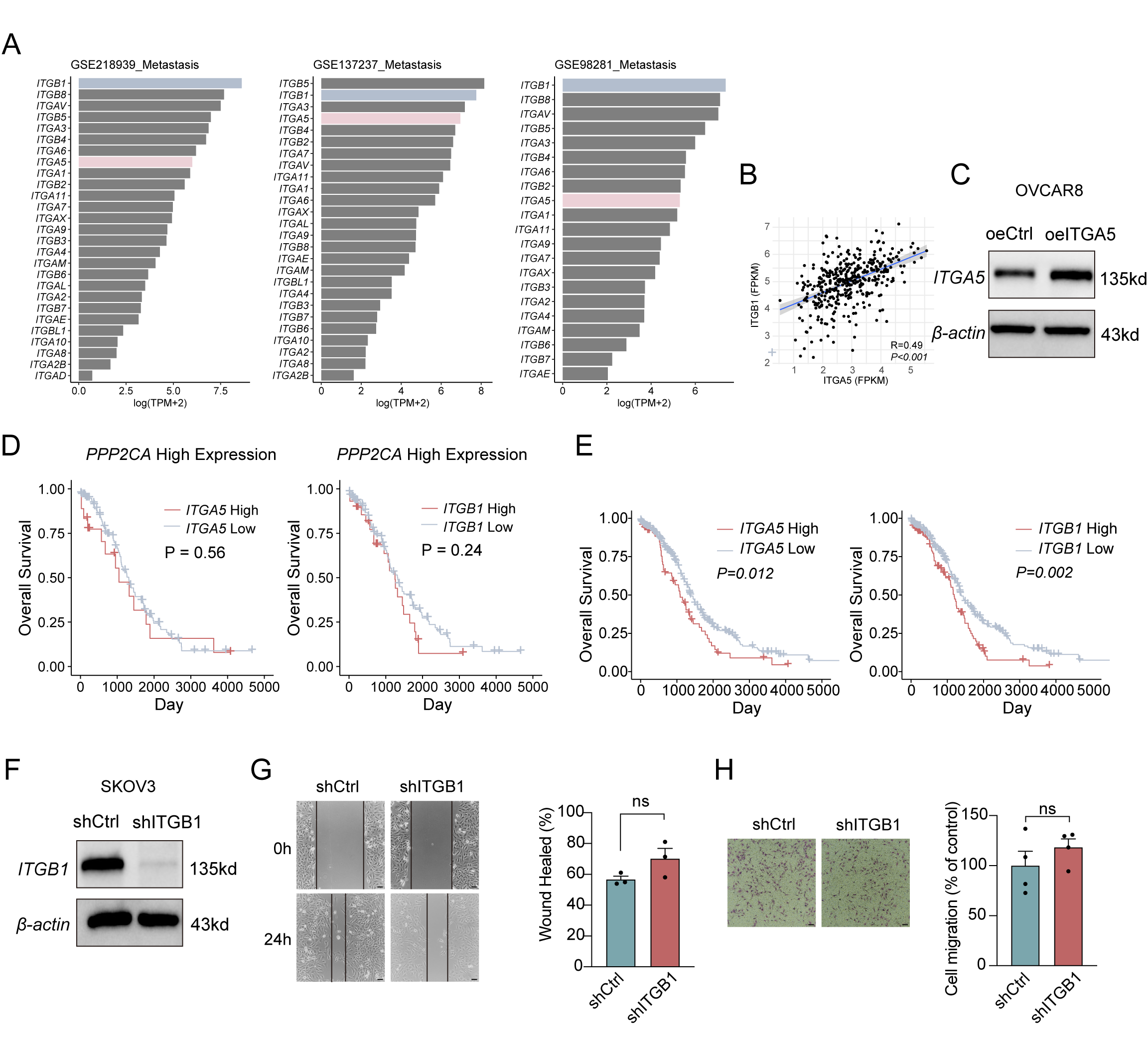


Figure S2. (A) Analysis of integrin family expression in OC metastases from the GEO dataset. (B) Correlation analysis between *ITGA5* and *ITGB1* expression in OC patients. (C) Western blot validation of *ITGA5*- overexpressing OVCAR8 cells. (D) Kaplan-Meier analysis of overall survival between the high and low expression groups of *ITGA5* and *ITGB1* among OC patients with high *PPP2CA* expression. (E) Overall survival analysis of *ITGA5* and *ITGB1* in OC patients. (F) Western blot validation of *ITGB1* knockdown. (G-H) Wound healing assay (G) and transwell assay (H) were performed on *ITGB1*-knockdown cells.


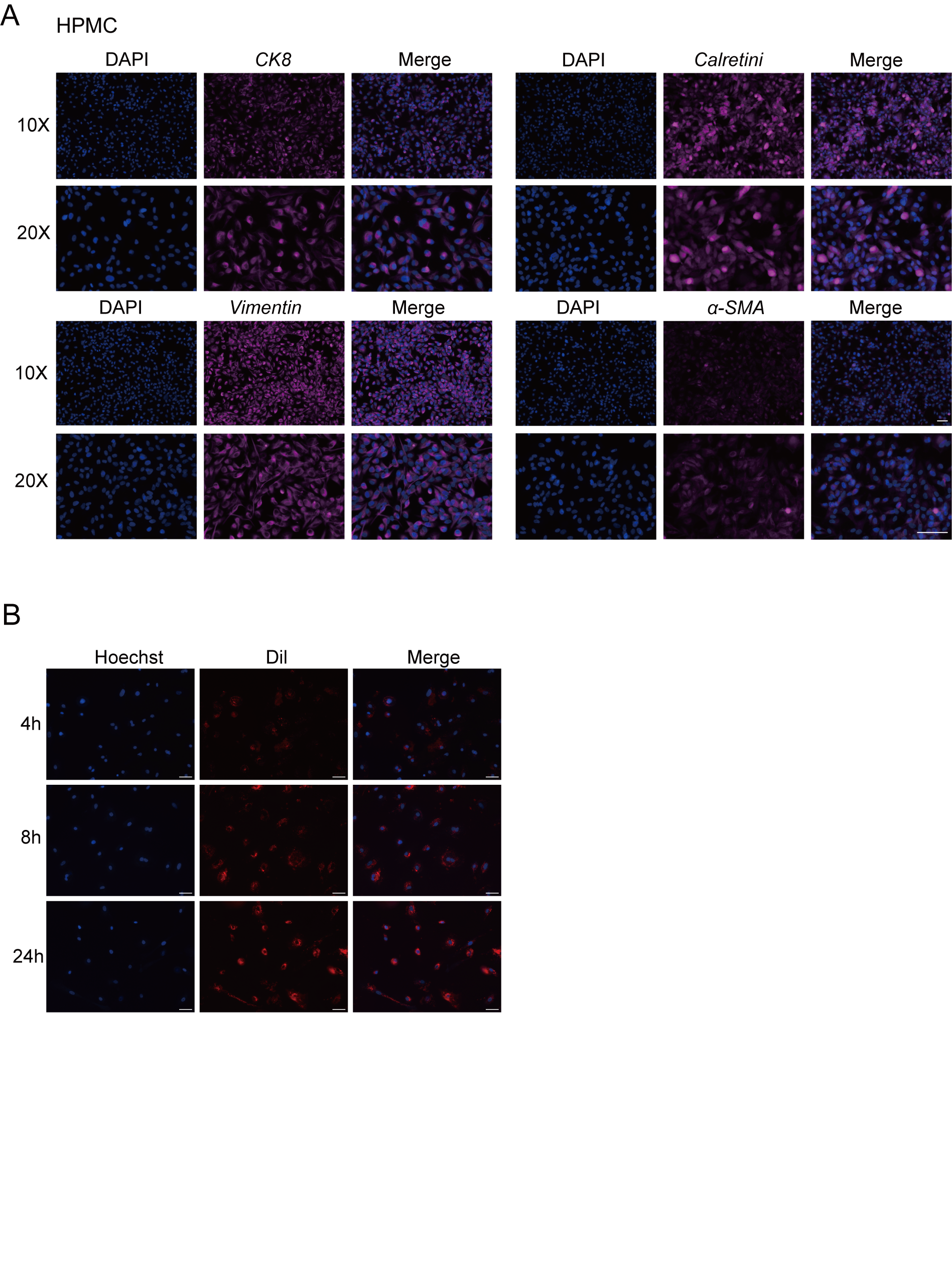


Figure S3. (A) Immunofluorescence characterization of HPMC revealed the positive expression of several markers, including *CK8*, *vimentin*, and *calretinin*, as well as the negative expression of the *α-SMA*. (B) Fluorescence microscopy imaging of mesothelial cells incubated with Dil-labeled SKOV3 exosomes at different time points (4 h, 8 h, 24 h), with Hoechst-stained nuclei. Scale bar = 50 µm.


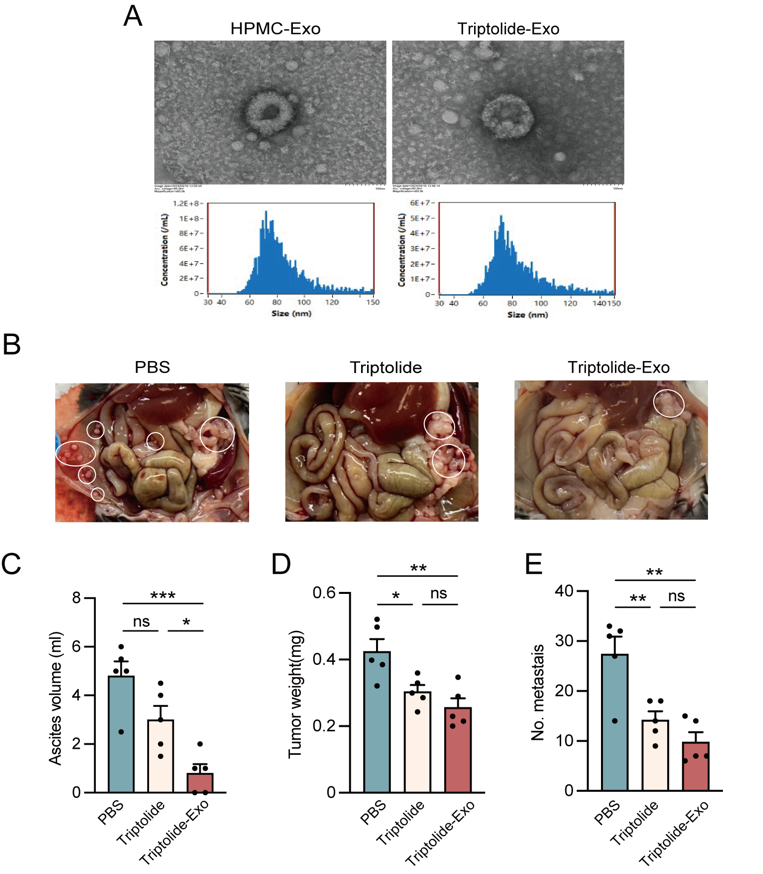


Figure S4. (A) Characterization of HPMC-derived exosome (HPMC-Exo) and Triptolide-loaded exosome (Triptolide-Exo) by transmission electron microscopy and nanoparticle size distribution analysis. (B) Establishment of an intraperitoneal OC model by injecting 2 × 10⁶ ID8 cells suspended in PBS into the peritoneal cavity of 6–8-week-old female C57BL/6 mice (n = 5). Representative images of intraperitoneal tumor formation in mice treated with PBS, free Triptolide, or Triptolide-Exo. (C–E) Quantification of ascites volume (C), tumor weight (D), and the number of metastatic nodules (E) in each treatment group. ns, not significant; *p < 0.05, **p < 0.01, ***p < 0.001.


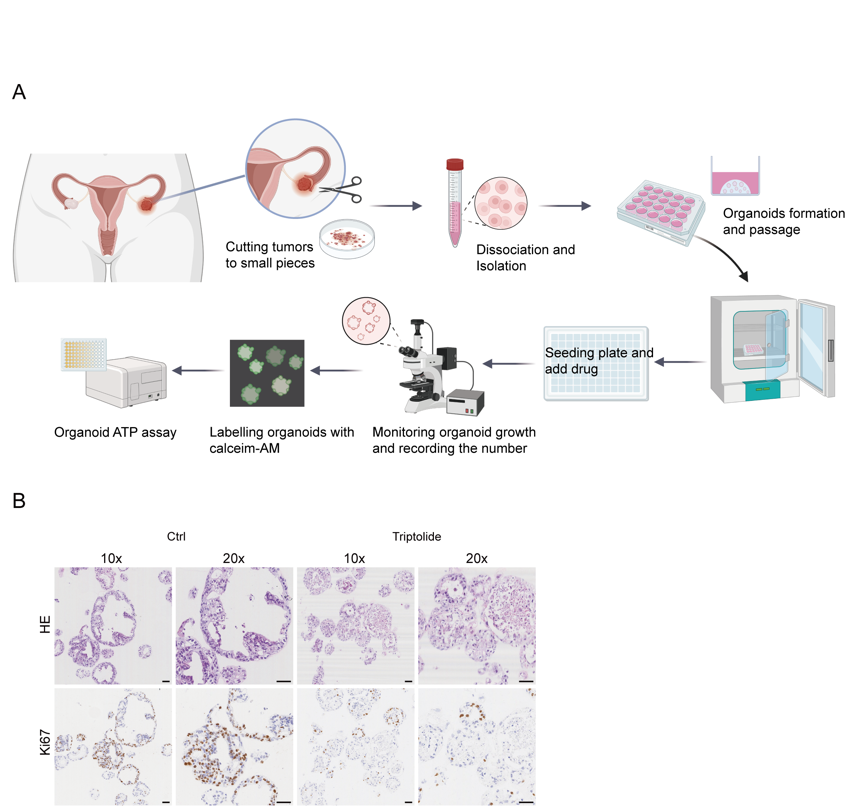


Figure S5. (A) Schematic of OC organoid generation from patient tumor tissues and subsequent Triptolide treatment. Organoids were monitored using Calcein-AM and ATP luminescence assays. (B) H&E and *Ki-67* staining of Triptolide-treated OC organoids (#11). Scale bar = 50 µm.
